# Fault Diagnosis of Semiconductor Detectors Using a Radial Basis Function Neural Network with Bayesian Optimization and K-Means Clustering

**DOI:** 10.3390/s26196116

**Published:** 2026-09-26

**Authors:** Yuxi Xie, Rui Yan, Xiang Li, Junwei Shi, Heng Zheng, Wei Xu, Dongfan Gong, Songsong Tang, Yanliang Tan, Yuebing Xu

**Affiliations:** 1 College of Physics and Electronic Engineering, Hengyang Normal University, Hengyang 421008, China; 18434642808@163.com (R.Y.); sjw011214@163.com (J.S.); gmwxyh@163.com (H.Z.); 15323539362@163.com (W.X.); 17398716787@163.com (D.G.); hytyl@163.com (Y.T.); yuebingxu@hnu.edu.cn (Y.X.); 2 School of Physical Sciences, University of Science and Technology of China, Hefei 230026, China; tangss@mail.ustc.edu.cn; 3 College of Computer Science and Technology, Hengyang Normal University, Hengyang 421002, China; ludlows2022@163.com

**Keywords:** semiconductor detector, simulated fault severity, radial basis function neural network, Bayesian optimization, K-means, waveform representation

## Abstract

This study evaluates radial basis function neural networks (RBFNNs) for severity classification within predefined semiconductor-detector fault categories. The datasets contain simulated PN aging, RC circuit faults and radiation damage, together with three hybrid-fault constructions. Raw waveforms were partitioned before fitting feature transformations. A four-variant ablation over ten stratified partitions assessed Bayesian optimization (BO) of the hidden-layer size and radial basis width, and K-means placement of the centers. With training-fitted principal component analysis (PCA), the combined model achieved mean test accuracies of 97.67 ± 1.97% for PN aging and 100.00 ± 0.00% for RC faults, but only 7.78 ± 1.40% for the 66-class radiation task. An exploratory radiation reassessment using pulse-amplitude and residual-noise features, with a distance-scaled RBF width, achieved 72.42 ± 1.57% accuracy and 72.26 ± 1.63% macro-F1 over three partitions. Three frozen radiation models achieved a mean accuracy of 71.83% on 66,000 newly generated waveforms. Three PN aging–RC partitions gave 86.40 ± 1.52% accuracy within the standard search range. A separately selected expanded-range model achieved 96.33% on 110,000 new simulated waveforms. And three PN aging–radiation damage and RC–radiation damage partitions gave 77.40 ± 1.38% and 78.45 ± 1.28% accuracy with the standard search range, respectively.

## 1. Introduction

Semiconductor radiation detectors convert deposited energy into electrical signals and are used in radiation measurement and monitoring [1,2]. Changes in charge collection and readout electronics can alter pulse amplitude, shape and noise. These changes motivate waveform-based condition monitoring, but a diagnostic label must be distinguishable from other conditions in the available signal. A model that separates selected simulated parameter levels therefore requires additional evaluation before it can support diagnosis of physical detector degradation.

Machine learning has been investigated for nuclear pulse analysis and detector monitoring. PCA-based pulse shape discrimination and support-vector approaches provide established ways to represent and classify pulse variation [3,4]. Pulse discrimination and fault diagnosis nevertheless have different targets: separation of waveform populations does not alone establish the identity of the physical degradation mechanism. The evaluation must therefore address both the classifier and the construction of its labels.

Prior scintillation-detector studies have used extreme learning machines [5], wavelet-packet features with multiclass support vector machines [6], and back-propagation neural networks [7]. A recent CZT detector study compared deep residual shrinkage networks and extreme learning machines [8]. These studies motivate nonlinear waveform classification. Their reported accuracies are not directly comparable across different data constructions, label definitions and evaluation protocols. For severity classification, classwise precision and recall, variability across partitions and the independence of preprocessing are needed alongside overall accuracy.

An RBFNN represents nonlinear relationships through localized Gaussian basis functions. Its behavior depends on the number, locations and widths of the functions and on the fitted output weights. Neural-network optimization and RBF-based monitoring have been examined in photovoltaic and motor applications [9,10,11]. These applications motivate evaluating RBF configuration choices for detector waveforms; they do not establish a general advantage over other classifiers.

BO guides hyperparameter evaluations through a surrogate model and an acquisition rule. Applications in reactor monitoring, turbine diagnosis and hydraulic-pump diagnosis illustrate its use with neural classifiers [12,13,14]. Cross-domain diagnosis methods, including KMDSAN [15], address the additional problem of a change in operating domain. KMDSAN addresses cross-domain bearing diagnosis [15], whereas domain-adversarial training uses labeled source observations and unlabeled target observations to learn a representation [16]. The present RBF classifier does not contain a domain-alignment objective. Additional samples from the same generator evaluate new waveform realizations; they do not establish transfer to measured detector signals.

Optimized RBF networks have also been studied for analog-circuit diagnosis [17]. In the present model, BO selects the number and width of the basis functions, whereas K-means locates their centers in the training-feature distribution. A four-variant ablation is used to separate their empirical contributions.

The contribution is an application-level reassessment of optimized RBFNNs for simulated detector-fault severity classification. The study provides a training-fitted preprocessing protocol, a controlled BO/K-means ablation, and a radiation-specific amplitude/noise representation. Single-fault categories and hybrid categories are treated as separate tasks. The results do not evaluate one classifier that identifies all physical fault types from an unknown operating state.

## 2. Methodology

### 2.1. Waveform Classification Framework

Each sample is a 300-point voltage waveform. For each single-fault task, 100 waveforms per class were stratified into 70 training, 15 validation and 15 test samples. Partitioning preceded all fitted preprocessing. Training data determined the scaling parameters, PCA directions or pulse template, RBF centers and output weights. Validation data determined hyper-parameters and the retained training epoch. Predictions for the designated test partition were obtained after the run configuration was fixed. Figure 1 distinguishes the initial PCA evaluation from the subsequent radiation-specific reassessment; the development history of the latter is described in Section 2.7.

### 2.2. Simulation of Semiconductor Detector Output Signals

The principle of semiconductor detectors is that when high-energy particles (such as γ-rays, X-rays, or β-particles) enter the depletion region, these particles generate electron-hole pairs through ionization. These electrons migrate towards the anode under the interaction of the electric field, generating a transient current. This current is processed by a charge-sensitive preamplifier and then converted into a voltage signal, ultimately enabling the counting and quantification of radiation energy. This paper takes CdZnTe detectors, for instance, and the radiation source used is ^137^Cs (the emission rate of γ photons = A (activity) × branching ratio = 3.7 × 10^10^ Bq × 0.85 = 3.15 × 10^10^ photons/second).

A double-exponential response with additive noise represents each sampled waveform. Analytical pulse modeling for semiconductor readout provides the general context [18]; the following parameterization defines the numerical signals evaluated here:

(1) $$x(t)=2A(e^{-t/\tau_{s}}-e^{-t/\tau_{f}})+\varepsilon (t)$$

Here *x*(*t*) is voltage, *A* is the pulse coefficient, *τ_f_* and *τ_s_* are the fast and slow time constants, and *ε*(*t*) is independent zero-mean Gaussian noise with standard deviation σ_n_. The amplitude coefficient varies between pulses as A~N(H, (0.015H/2.355)^2^), where H is the class-specific mean amplitude coefficient. H is a scale coefficient of the double-exponential response and is not its sampled peak voltage.

In actual fault conditions, it is difficult to fully acquire nuclear signal data covering all fault types and different severity levels; moreover, the experimental costs are high. To address this, this paper adopts a method that combines measured with simulated data. According to the features of measured signals under normal conditions, the parameters of the simulated signal model are set as follows: the average pulse amplitude is 1 V, the standard deviation of the white noise is 0.0005 V, the decay time constants of the fast and slow components are 20 and 90 respectively, and the energy resolution is 0.015. Figure 2 presents a comparison between the actual measured signal and the simulated signal under normal conditions. This study focuses on the features of the waveform before attenuation. Therefore, baseline fluctuations are not taken into account. And the baseline at the trailing end of the waveform is smoothed while simulating the signal under normal conditions.

### 2.3. Classification of Semiconductor Detector Fault Types

The three single-fault datasets contain 100 waveforms per parameter-defined class: 10 PN aging classes (1000 waveforms), 11 RC classes (1100 waveforms), and 66 radiation classes (6600 waveforms). The hybrid inputs contain 110 PN–RC classes (11,000 waveforms), 396 RC–radiation classes (39,600 waveforms), and 360 PN–radiation classes (36,000 waveforms). All 95,300 prepared waveforms contain finite values, and no exactly identical complete waveforms were found within or across these six prepared tasks. For the normal state, this paper generates 500 sets of measured data and 500 sets of simulated data.

PN aging is parameterized by ten mean amplitude coefficients—H = 0.35, 0.40, …, 0.80 V—with the normal time constants and baseline noise. The levels represent reduced signal amplitude as a proxy for impaired charge collection. Earlier scintillation-detector work [6] motivates waveform-based fault parameterization, but it does not calibrate these selected amplitude levels as physical CZT aging thresholds.

RC faults vary the slow time constant as *τ_s_* = 90 ns while keeping *τ_f_* = 20 ns. The eleven modeled ratios are r = 1/8, 1/7, 1/6, 1/5, 1/4, 4, 5, 6, 7, 8 and infinity; the infinite limit makes the slow exponential equal to one over the sampled interval. These settings define a parameter study of pulse-shape changes. Analog-circuit soft-fault diagnosis [19] provides related context but does not establish these ratios as calibrated CZT fault thresholds.

Radiation damage is represented by eleven mean amplitude coefficients—H = 0.35, 0.40, …, 0.85 V—and six noise multipliers—q = 100, 110, 120, 130, 140 and 150, with σ_n_ = 0.0005q V. Their Cartesian product defines 66 joint amplitude–noise classes. For example, label 35100 denotes H = 0.35 V and q = 100. The time constants remain 20 ns and 90 ns. Radiation effects in semiconductor devices provide general motivation for signal degradation [20]; the numerical amplitude and noise ranges used here remain chosen model conditions, without measured calibration to CZT radiation dose.

The fault names identify the intended modeled scenarios. An amplitude reduction alone is not unique to junction aging, and increased white noise is not a complete model of radiation damage. The task therefore measures discrimination among specified parameter labels within each scenario.

### 2.4. Training-Fitted Waveform Representations

The initial ablation uses one shared PCA coordinate system per training partition. PCA is an established compression method for monitoring signals [21]. Let *X* contain the *N* training waveforms, each with p = 300 samples:

(2) $$X\in R^{N\times 300},X_{j,:}=x_{j}^{T}$$

Each row *x_j_* is one waveform. All normalization statistics below are estimated from these *N* training rows; validation and test rows are transformed without refitting.

(3) $$Z_{jl}=\frac{X_{jl}-\mu_{l}}{s_{l}},j=1,\ldots ,N,l=1,\ldots ,300$$

The pointwise training mean *μ_l_* and sample standard deviation *s_l_* define Equation (3); a zero standard deviation is replaced by one. The normalized matrix is centered by its training mean before covariance decomposition. Eigenvalues are sorted in descending order and the smallest d explaining at least 99% of the training variance is retained:

(4) $$S=\frac{\left(Z-\bar{Z}{)}^{T}(Z-\bar{Z}\right)}{N-1}$$

(5) $$SV=V\Lambda ,\lambda_{1}\geq \lambda_{2}\geq \ldots \geq 0$$

(6) $$d=min\{k:\frac{\sum_{l=1}^{k}\lambda_{l}}{\sum_{l=1}^{300}\lambda_{l}}\geq 0.99\}$$

(7) $$F=(Z-\bar{Z})V_{d}$$

*V_d_* contains the first d eigenvectors. After projection, each score coordinate is mapped using its training minimum and range; a zero range is replaced by one. The same affine map is applied to held-out scores without clipping. This procedure retains 15, 2 and 277 components for PN, RC and radiation, respectively, in every one of the ten evaluated partitions. Table 1 reports the training-set cumulative explained variance at the requested fixed dimensions.

For the radiation reassessment, a two-dimensional representation replaces PCA. The mean training waveform is normalized to unit Euclidean norm to obtain a template u. For a waveform x, the features are a = x^T^u and log(r), where r = sqrt(||x − au||^2^/300). The residual RMS is floored at the numerical epsilon determined from training amplitudes before taking the logarithm. Both features are standardized using their training means and sample standard deviations. This construction represents pulse amplitude and residual noise explicitly; it is applied only to the radiation reassessment.

### 2.5. RBFNN Configuration and Training

#### 2.5.1. Gaussian RBF Classifier

The classifier maps a feature vector f of dimension d to C class probabilities through K Gaussian basis functions and a linear softmax output. Figure 3 identifies the fitted components. The centers c_i_ and shared width σ determine the hidden responses; W contains the K × C output weights. The implemented output layer has no additive bias.

The conventional RBF and BO-only variants select centers from training feature vectors without replacement. The K-means variants use training-cluster centroids. Conditional on these centers and the shared width, full-batch Adam updates only W. The nonlinear basis is therefore fixed throughout each candidate training run.

For hidden unit *i*, the Gaussian response is

(8) $$h_{i}(f)=exp(-\frac{\|f-c_{i}{\|}^{2}}{2\sigma^{2}}),i=1,\ldots ,K$$

where *c_i_* is a d-dimensional training-derived center and *σ* > 0 is the common width. The class-k logit is the weighted sum of the hidden responses:

(9) $$z_{k}(f)=\sum_{i=1}^{K}W_{ik}h_{i}(f),k=1,\ldots ,C$$

*W_ik_* connects hidden unit *i* to class *k*. No bias term is included in Equation (9), matching the evaluated implementation. Softmax converts the logits to probabilities:

(10) $$p_{k}(f)=\frac{exp(z_{k}(f))}{\sum_{l=1}^{C}exp(z_{l}(f))}$$

For N training samples and one-hot labels *y_jk_*, the loss is mean cross-entropy:

(11) $$L=-\frac{1}{N}\sum_{j=1}^{N}\sum_{k=1}^{C}y_{jk}logp_{k}(f_{j})$$

The predicted probability for sample *j* and class *k* is *p_jk_*. Adam [22] uses the loss gradient *g_t_* to update first and second moments, with elementwise multiplication and division:

(12) $$\begin{array}{c} m_{t}=\beta_{1}m_{t-1}+(1-\beta_{1})g_{t}\\ v_{t}=\beta_{2}v_{t-1}+(1-\beta_{2})(g_{t}⊙g_{t})\\ W_{t}=W_{t-1}-\eta \frac{{\hat{m}}_{t}}{\sqrt{{\hat{v}}_{t}}+\epsilon } \end{array}$$

Bias-corrected moments are m^_t_ = m_t_/(1 − β_1_^t^) and v^_t_ = v_t_/(1 − β_2_^t^). The learning rate is 0.01, β_1_ = 0.9, β_2_ = 0.999 and ε = 10^−8^. Weights start from N(0, 0.01^2^). Every epoch uses all training samples. Training stops after at most 2000 epochs or 50 epochs without a validation-loss improvement exceeding 10^−6^; the weights with the lowest observed validation loss are retained. Centers and widths are not updated by Adam.

#### 2.5.2. The Selection of Hyper-Parameters with BO

BO minimizes validation misclassification rate over K and the width parameter [23]. In the PCA ablation, σ is searched directly on a logarithmic scale over 0.1–20. For the radiation-specific features, σ = ασ_ref_, with α searched logarithmically over 0.1–3. Here σ_ref_ is the median positive pairwise distance among up to 512 evenly spaced training rows divided by sqrt(2 ln 2). Thus α = 1 makes the Gaussian response at this median distance equal to 0.5. Table 1 defines both search spaces.

The Bayesian optimization implementation uses a Gaussian-process surrogate with an automatic-relevance-determination Matérn 5/2 covariance kernel. The surrogate approximates the validation objective *L*_val_ as a function of the candidate *θ* = (K, σ) or (K, α):

(13) $$L_{val}(\theta )\sim GP(m(\theta ),\kappa (\theta ,\theta^{\prime }))$$

The implemented acquisition rule is expected-improvement-plus. Ordinary expected improvement for a minimization objective has the form in Equations (14) and (15); the plus variant adds exploration adjustment.

(14) $$EI(\theta )=(L_{best}-\mu (\theta ))\Phi (z)+s(\theta )\phi (z)$$

(15) $$z=\frac{L_{best}-\mu (\theta )}{s(\theta )},s(\theta )>0$$

*L*_best_ is the lowest observed validation error, *μ*(*θ*) and *s*(*θ*) are the surrogate predictive mean and standard deviation, and *Φ* and *φ* are the standard normal cumulative distribution and density. At zero predictive variance, the limiting improvement is used. Each search evaluates 30 candidates in total, including five initial points. The best observed finite validation objective is selected. Table 2 gives the executed sequence.

#### 2.5.3. Determination of the Center Values of Conventional RBFNN with K-Means

K-means uses k-means++ initialization, three restarts, a maximum of 1000 iterations and a convergence tolerance of 10^−6^. Empty clusters use the singleton option. The restart with the smallest within-cluster squared-distance objective supplies the centers. This procedure seeks a local minimum and does not establish a globally optimal center configuration.

For each training feature vector *f_j_*, its assigned cluster *a_j_* is determined by the nearest center:

(16) $$a_{j}=\underset{i=1,\ldots ,K}{argmin}\|f_{j}-c_{i}{\|}^{2}$$

After assignment, each center is the mean of the feature vectors assigned to its cluster:

(17) $$c_{i}=\frac{1}{\left|S_{i}\right|}\sum_{j\in S_{i}}f_{j}$$

*S_i_* is the set of training indices assigned to cluster *i*. For split seed *s*, each candidate fit uses seed *s* + 10,000; the BO search uses seed *s* + 11,000. The model random state is reset for each candidate and the search state is restored afterward. Repeated evaluation of an identical candidate is therefore deterministic under the specified implementation. All four ablation variants use the same split and preprocessing.

### 2.6. Evaluation Metrics

Let *M* be the *C* × *C* confusion matrix, with true classes in rows and predicted classes in columns. Overall accuracy is the diagonal sum divided by the total number of evaluated waveforms. Precision and recall are computed separately for each class:

(18) $$Accuracy=\frac{\sum_{k=1}^{C}M_{kk}}{\sum_{j=1}^{C}\sum_{k=1}^{C}M_{jk}}$$

(19) $$R_{k}=\frac{M_{kk}}{\sum_{l=1}^{C}M_{kl}}$$

(20) $$P_{k}=\frac{M_{kk}}{\sum_{l=1}^{C}M_{lk}}$$

A zero precision denominator is assigned precision zero. Per-class F1 is 2*PkRk*/(*P_k_* + *R_k_*), with zero assigned when both terms are zero. Macro-precision, macro-recall and macro-F1 average the corresponding *C* class values equally. Results are then summarized across runs as mean ± sample standard deviation. These are descriptive variations over partitions of the same finite dataset, not confidence intervals from independent detector experiments. Each class has equal test support within its task, so macro-recall equals overall accuracy.

### 2.7. Computational Evaluation Protocol

Figure 4 presents the hardware block diagram of the measurement and simulation platform in this study. The PCI-9846 (Adlink Technology (Shanghai China) Co., Ltd.) high-speed data acquisition card is adopted to collect the normal output signals of the CZT detector, which are regarded as the measured normal-state signals. These signals are stored on the host computer in .csv format and can then be read via the PyCharm platform (2024.2.1). Based on the normal state and the mathematical model shown in Equation (1), four types of nuclear pulse signals (including normal state, PN junction aging fault, RC fault, and radiation damage fault) are generated through simulation on the PyCharm platform. The initial evaluation used ten stratified partitions with random seeds 2026–2035. Each partition was shared by conventional RBF, K-means RBF, BO RBF and BO + K-means RBF. The two variants without BO used K = 50 and σ = 1. The PyCharm ran on an Intel Core i5-14600KF CPU with six computational threads. Single-pulse predictions were recorded for the held-out partitions.

The radiation feature reassessment followed inspection of the initial PCA results. Candidate feature and width configurations were selected using validation results on seeds 2026–2028; the chosen configuration was fixed before its final test predictions were computed. These test partitions reuse three historical splits and were not newly acquired, untouched external data. The revised radiation result is therefore reported as an exploratory reassessment with three runs, separately from the ten-run PCA ablation. Subsequently, the three saved radiation models and their preprocessing were frozen and evaluated on 66,000 newly simulated waveforms, 1000 per class, generated with seed 2026091201. Model hashes were recorded before generation. No parameter was fitted on these additional samples. The models share the new evaluation set; their variation is not variation between independent detector experiments.

## 3. Results

### 3.1. PCA Representation and Four-Variant Ablation

Training-fitted PCA produces markedly different dimensionalities across tasks (Table 3). PN and RC reach 99% explained variance using 15 and two components, whereas radiation requires 277. At 250 components, radiation retains 97.31% of training variance. The four-variant results in Figure 5 show that BO raises PN mean test accuracy from 80.27% to 95.73%, and combining BO with K-means gives 97.67%. K-means alone reaches 85.53%. RC is already nearly perfectly separated by the baseline (99.58%) and reaches 100% with each modified variant. Radiation remains poorly classified with this PCA representation: the four respective accuracies are 4.27%, 7.80%, 4.87% and 7.78%.

An additional PN–RC validation-only dimension check used fixed K = 50 K-means centers and width σ = 1. At 50, 100, 150, 200 and 250 PCs, the three-partition validation accuracies were 38.51 ± 2.46%, 31.37 ± 3.77%, 26.87 ± 2.15%, 25.05 ± 0.71%, and 21.47 ± 1.46%, respectively. Even 50 PCs explained more than 99.9998% of training variance. Test accuracy was not used to select the dimension.

### 3.2. Radiation-Specific Representation and Model Training

Explicit amplitude and residual-noise features improved the radiation validation results. With K = 50 and a relative width factor of one, the mean validation accuracy over three partitions was 70.74%; BO with K-means increased it to 71.14%. The selected numbers of centers were 64, 94 and 26, with relative width factors 0.4452, 0.5591 and 0.6594 for seeds 2026, 2027 and 2028, respectively. The corresponding actual widths were 0.6628, 0.8337 and 1.0085. Selected parameters for the completed combined-model evaluations are reported in Table 4.

Figure 6 reports the saved training and validation trajectories for these three radiation models. All three retained epoch 2000. Their final training accuracies were 72.53%, 72.88% and 73.03%, while validation accuracies were 71.92%, 70.51% and 71.01%. Neither loss approached zero, nor did accuracy approach 100%. A further validation-only comparison of a whitened output basis with an output bias and L-BFGS-B optimization gave mean validation accuracies of 70.03% without a penalty and 70.67% with a 0.001 penalty. It did not improve on the selected Adam model, which was retained for final evaluation.

### 3.3. Single-Fault Severity Classification

The PN, RC and radiation results refer to three separate severity-classification problems. PN and RC use the completed ten-run PCA protocol; the revised radiation result uses the three-run amplitude/noise reassessment. Table 5 gives accuracy and macro-averaged metrics with their run-to-run variation. These task-specific results are not pooled into a single “single-fault accuracy” because the numbers of classes, difficulty and number of evaluated partitions differ.

For PN aging, the combined model achieves 97.67 ± 1.97% accuracy, 97.82 ± 1.84% macro-precision and 97.68 ± 1.96% macro-F1. For RC, accuracy, macro-precision, macro-recall and macro-F1 are all 100.00 ± 0.00% over the evaluated splits. Figure 7 presents classwise precision and recall. Perfect RC performance describes the selected simulated ratios and does not establish performance between those ratios or under a different readout response.

The revised 66-class radiation models correctly classify 735, 708 and 708 of 990 test waveforms, giving accuracies of 74.24%, 71.52% and 71.52%. The mean is 72.42 ± 1.57%, with macro-precision 73.05 ± 1.80% and macro-F1 72.26 ± 1.63%. Figure 8 shows the aggregated confusion matrix and recall by noise level. Repeated test appearances across partitions are repeated evaluations of the same finite dataset; the sum of their supports is not an independent sample count.

On the new 66,000-waveform set, the seed-2026, seed-2027 and seed-2028 radiation models correctly classified 47,451, 47,389 and 47,377 samples, respectively. Their accuracies were 71.90%, 71.80% and 71.78%, with a mean of 71.83%.

The radiation result is consistent with substantial overlap between adjacent noise levels. Under the stated independent Gaussian generator and equal class priors, a known-parameter classifier evaluated by Monte Carlo sampling gives 72.25% expected joint-label accuracy (1,320,000 draws; simulation uncertainty approximately ±0.075 percentage points at 95%). Even if the true amplitude and its label are supplied, the analytical expected accuracy for six noise levels from 300 points is 72.99%. These are conditional population references for the specified generator, not guarantees for arbitrary waveform files. They explain why matching feature scale and RBF width improves classification.

### 3.4. Hybrid-Fault Severity Classification

The PN aging–RC task was evaluated over three partitions with seeds 2026–2028, using training-fitted PCA and 30 BO evaluations per partition. The standard search covered 10–100 centers and RBF widths 0.1–20, and gave 86.40 ± 1.52% test accuracy. These runs reached the upper center limit and widths near the lower search bound. A separate validation comparison of four prespecified combinations of 150 or 200 centers and widths 0.02 or 0.05 selected K = 200 and σ = 0.02. This selected model was frozen before a new 110,000-waveform set was generated. It correctly classified 105,960 samples (96.33%), compared with 96,299 (87.54%) for the frozen standard-range seed-2026 model on the same new set. The expanded-range result is a separate single-model evaluation and does not replace the three-run standard-range mean. Macro-precision, macro-recall and macro-F1 for the three standard-range partitions were 86.91 ± 2.02%, 86.40 ± 1.52%, and 84.89 ± 1.72%, respectively.

The PN aging–radiation damage and RC–radiation damage tasks are also evaluated across three partitions, with 30 BO evaluations conducted in each partition. Unlike the PN aging–RC task, the radiation damage component in these two aforementioned tasks employs the amplitude-noise method, and the test accuracies are 77.40 ± 1.38% and 78.45 ± 1.28% respectively. For the PN aging–radiation damage task, the macro-precision, macro-recall, and macro-F1 of the three standard-range partitions are 78.33 ± 1.45%, 77.40 ± 1.38%, and 76.31 ± 1.65% respectively. For the RC–radiation damage task, the macro-precision, macro-recall, and macro-F1 of the three standard-range partitions are 79.25 ± 1.56%, 78.45 ± 1.28%, and 77.67 ± 1.94% respectively. Table 6 gives accuracy and macro-averaged metrics with their run-to-run variation.

Compared with single faults, hybrid faults increase the classification difficulty due to feature aliasing or nonlinear coupling. Figure 9, Figure 10 and Figure 11 respectively present the test set classification results of three types of faults with varying levels, namely PN aging–RC, PN aging–radiation damage and RC–radiation damage. (Recall, precision, and F1-score all fluctuate within the range of 40–100%.)

For the PN aging–RC task, PN aging faults mainly lead to a decrease in charge collection efficiency, thereby causing amplitude attenuation of pulse signals. RC faults are primarily manifested as changes in the time response characteristics of the front-end electronics system, including alterations in the rise time and fall time of pulses. Since the two faults act on the amplitude domain and time domain features of the signal respectively, they have relatively independent representations in the pulse waveform. The training and fitted PCA can retain the main information of amplitude changes and time response changes, enabling the faults to have strong separability in the feature space. As a result, the classification performance of most fault levels exceeds 70%, with only a few levels showing lower performance.

For the PN aging–radiation damage and RC–radiation damage tasks, radiation damage not only affects charge collection efficiency but also causes an increase in signal noise characteristics. The enhanced noise characteristics may mask the distinguishable features between radiation damage and PN aging in the amplitude domain (PN aging and radiation damage affect pulse amplitude) as well as the time-domain features induced by RC faults, resulting in blurred decision boundaries. Therefore, the classification performance of these two types of tasks fluctuates around the macro-average.

### 3.5. Comparisons of PN and RC Tasks

Table 7 compares completed three-partition evaluations using the same seeds, raw waveform assignments and training-fitted PCA inputs. Multi-SVM uses an RBF kernel and one-versus-one coding, with box constraint of 0.01–1000 and kernel scale of 0.01–100. BPNN uses one hidden layer with 10–100 units, cross-entropy, scaled conjugate-gradient training and regularization of 0–0.5. These SVM and BPNN searches each contain 30 BO evaluations, including five initial points; BPNN uses at most 2000 epochs and validation patience of 50. DRSN uses two residual shrinkage blocks, 8–64 channels, Adam learning rate of 0.0001–0.01, batch size of 32, at most 100 epochs and validation patience of 50. Its search stops at the first zero validation error or the 30-evaluation cap, retaining the earliest minimum. DRSN uses trainNetwork on an RTX 4060 Ti with fixed CPU/GPU seeds and deterministic GPU algorithms. Earlier SVM/BPNN runs use the recorded six-thread CPU setting; DRSN CPU operations use three threads. Timings describe these executions and are not a hardware-matched speed benchmark.

Multi-SVM and BPNN each achieved 100% test accuracy on all three PN and RC partitions. The completed DRSN PN and RC evaluations also achieved 100% on each partition. These comparisons do not establish a general superiority of the optimized RBF classifier. The results concern the specified simulated severity tasks.

A coding-scheme check on the same three PN partitions gave 51.56 ± 2.69% accuracy for one-versus-all SVM, whereas the one-versus-one SVM in Table 7 gave 100% on each partition. The stronger one-versus-one comparator is retained. For radiation, completed seed-2026 PCA-based comparator runs correctly classified 115 and 118 of 990 test waveforms with one-versus-all SVM and BPNN, respectively. Their accuracies were 11.62% and 11.92%, with macro-F1 values of 9.25% and 8.69%. These two radiation results each represent one partition, with 30 BO evaluations and model selection by validation error. BPNN used double-precision GPU training with the same architecture, search ranges and stopping criteria described above. They are not three-run estimates and do not provide a feature-matched comparison with the amplitude/noise RBF reassessment.

## 4. Discussion

The ablation supports a benefit of BO and K-means for PN severity classification within the evaluated simulation. RC is largely separated by its chosen pulse shapes, so the small improvement over the already strong baseline does not imply that optimization is equally important for every fault scenario. The initial radiation failure and the subsequent improvement show that an RBF width is meaningful only relative to the chosen feature representation and scale.

Specified disturbances were applied to new simulated radiation waveforms while the three saved models, their preprocessing and the target labels remained fixed. Each condition contained 66,000 waveforms. Controls A and B used independent generation seeds 2026091202 and 2026091301. The offsets, gain multipliers, correlation and second-pulse parameters are selected test conditions, not measured device limits. Gain changes alter the observed signal while retaining the original severity label, thereby testing nuisance gain shifts. Table 8 shows substantial sensitivity to these disturbances. No model was refitted after these results were inspected.

All formal classification results use simulated faults and closed-set severity labels already represented in training. They measure discrimination of new waveform realizations at the same nominal parameter levels. Holding out an entire severity class would remove that class from a closed-set classifier’s training labels; evaluating unseen severity therefore requires a defined interpolation, regression or open-set task.

The computational cost includes both the search and the final model fit. BO performs 30 candidate evaluations per partition, whereas the fixed variants perform one fit. The stored timing results are summarized in Table 9, with search and final training reported separately. Prediction uses one waveform at a time conceptually, although the recorded inference times concern batches of test waveforms. Table 7 gives the completed cross-model timings. None of these values includes acquisition, trigger or alarm latency.

## 5. Conclusions

A controlled RBFNN evaluation was completed for three separate simulated single-fault severity tasks. Training-fitted preprocessing, a four-variant BO/K-means ablation and explicit classwise metrics support a reproducible comparison of the evaluated configurations.

The combined configuration gives 97.67 ± 1.97% PN test accuracy and 100.00 ± 0.00% RC accuracy over ten partitions. The radiation-specific amplitude/noise representation gives 72.42 ± 1.57% accuracy over three historical partitions, an exploratory result consistent with overlapping noise-level distributions. The frozen radiation models achieved 71.83% mean accuracy on newly generated waveforms. The completed PN/RC comparator results do not support general RBF superiority. PN aging–RC accuracy was 86.40 ± 1.52% over three standard-range partitions; a separately selected expanded-range model achieved 96.33% on new simulated waveforms. And PN aging–radiation damage and RC–radiation damage accuracy was 77.40 ± 1.38% and 78.45 ± 1.28% over three standard-range partitions, respectively.

In this study, due to the limitations of current conditions, validation diagnostics for physical degradation in real CZT detectors cannot be conducted in the short term. We will carry out relevant validation experiments in the future.

## Figures and Tables

**Figure 1:**
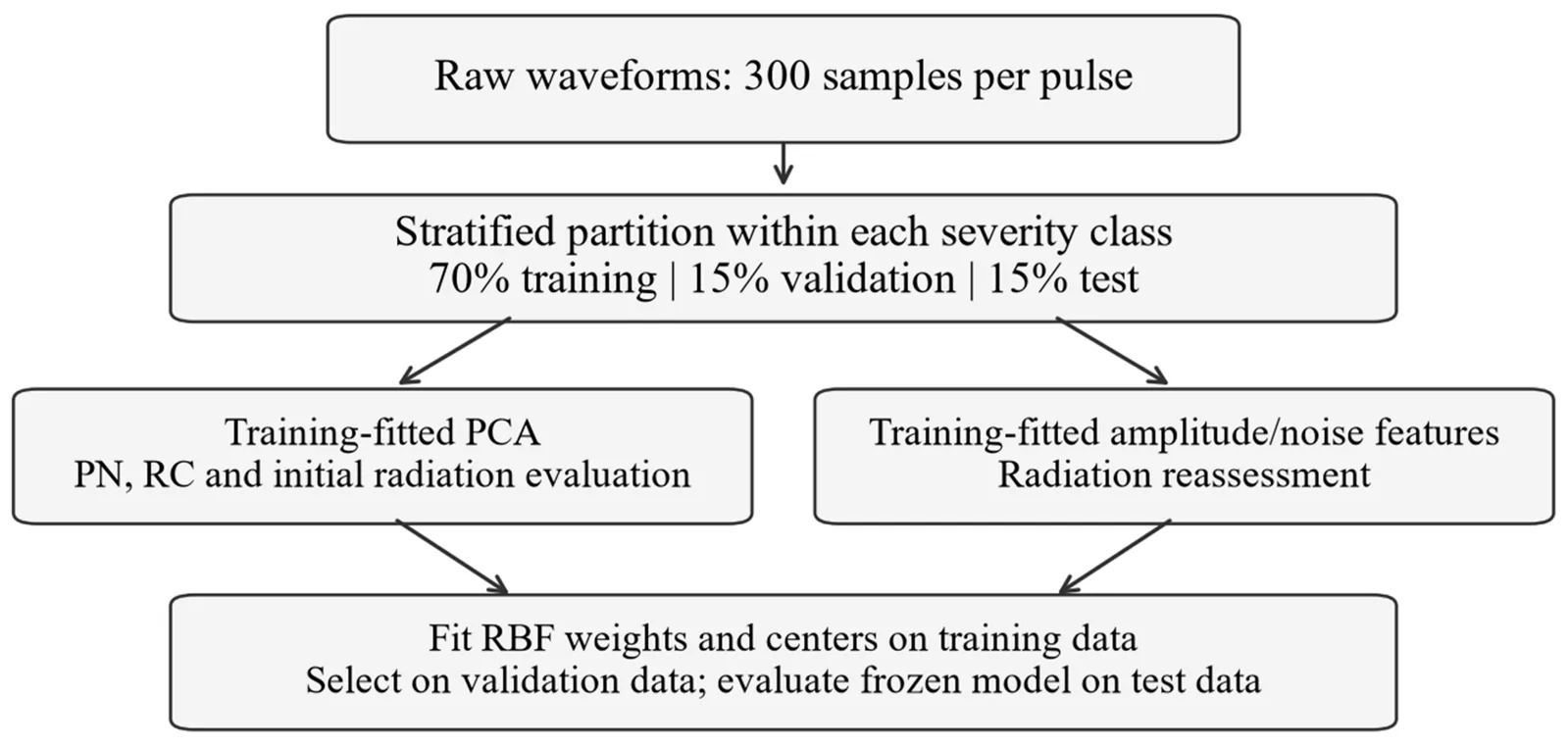
Evaluation sequence for the single-fault tasks. Feature transformations are fitted on the training partition and applied unchanged to the validation and test partitions.

**Figure 2:**
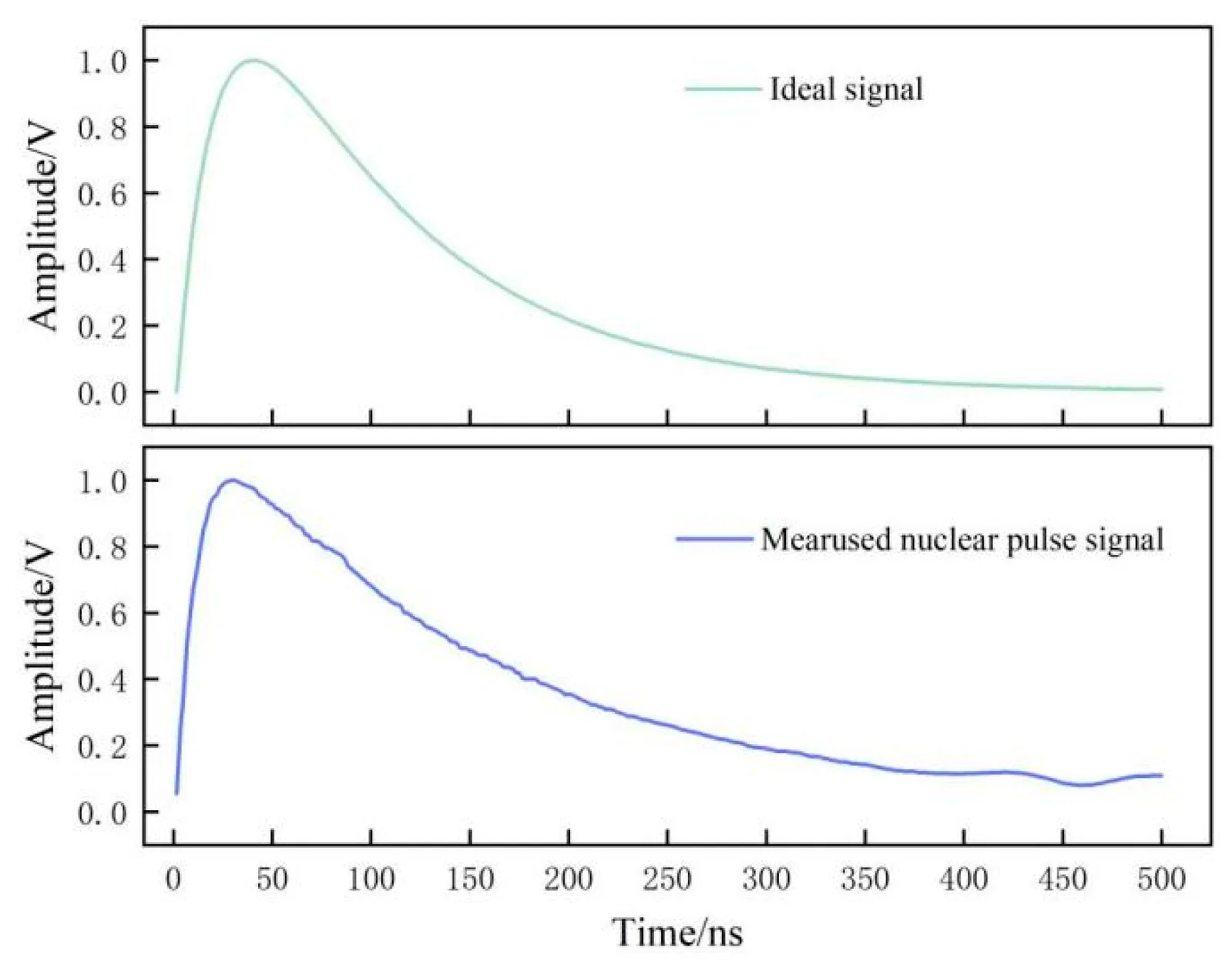
Comparison between actual measurement and simulated signal.

**Figure 3:**
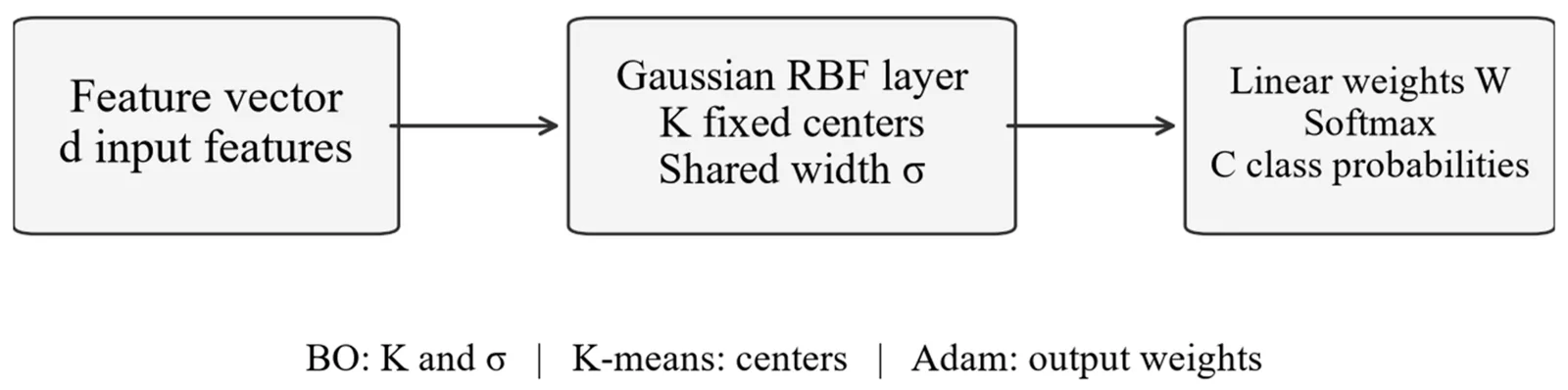
RBF classifier. The centers and width remain fixed during output-weight training; the combined configuration uses K-means centers and BO-selected K and σ.

**Figure 4:**
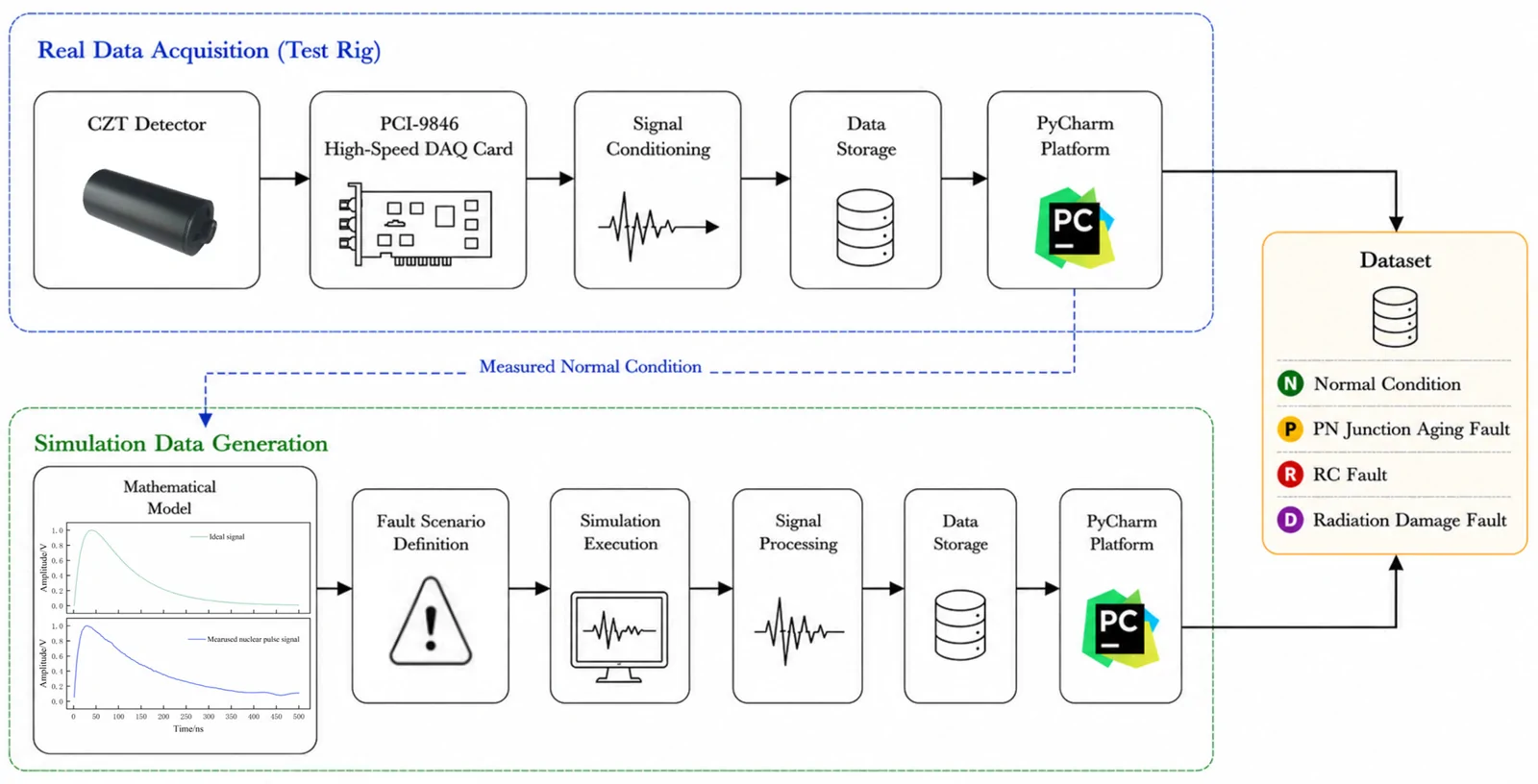
Hardware block diagram of the measurement and simulation platform.

**Figure 5:**
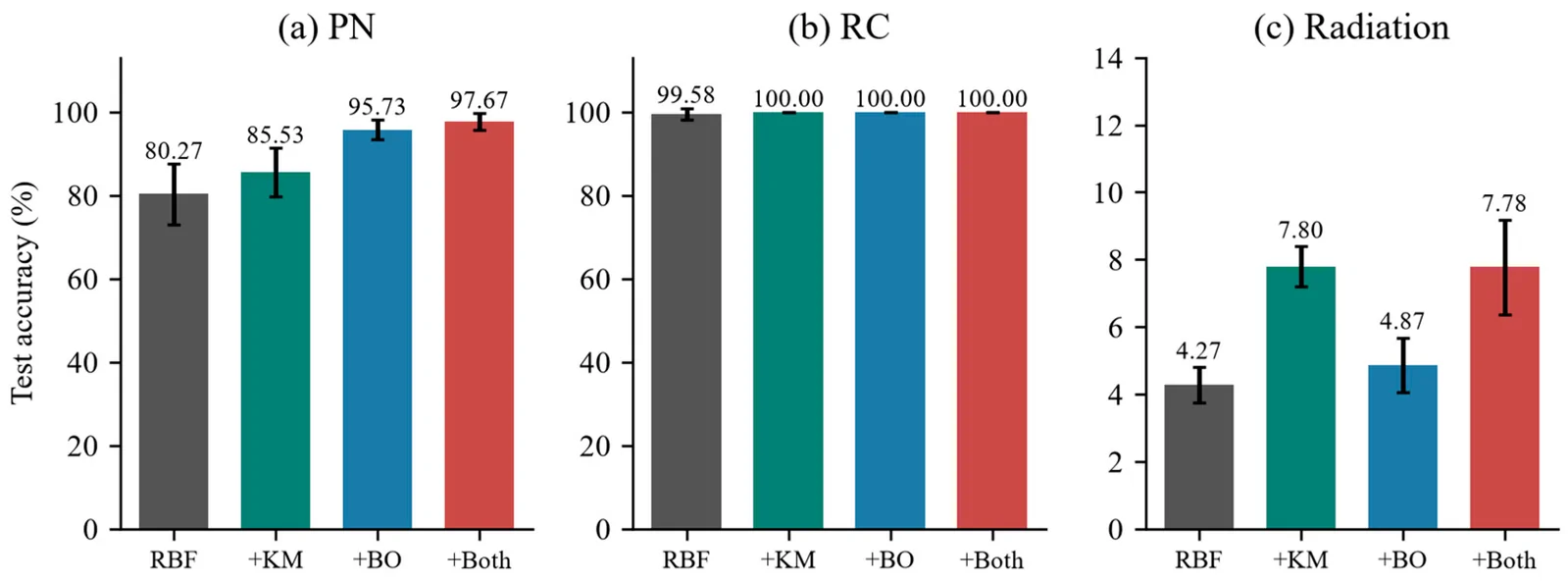
Test accuracy for the four RBF variants using the initial PCA representation: (**a**) PN, (**b**) RC and (**c**) radiation. Bars show means and error bars show sample standard deviations over ten shared partitions. KM denotes K-means. The radiation panel uses a different vertical scale.

**Figure 6:**
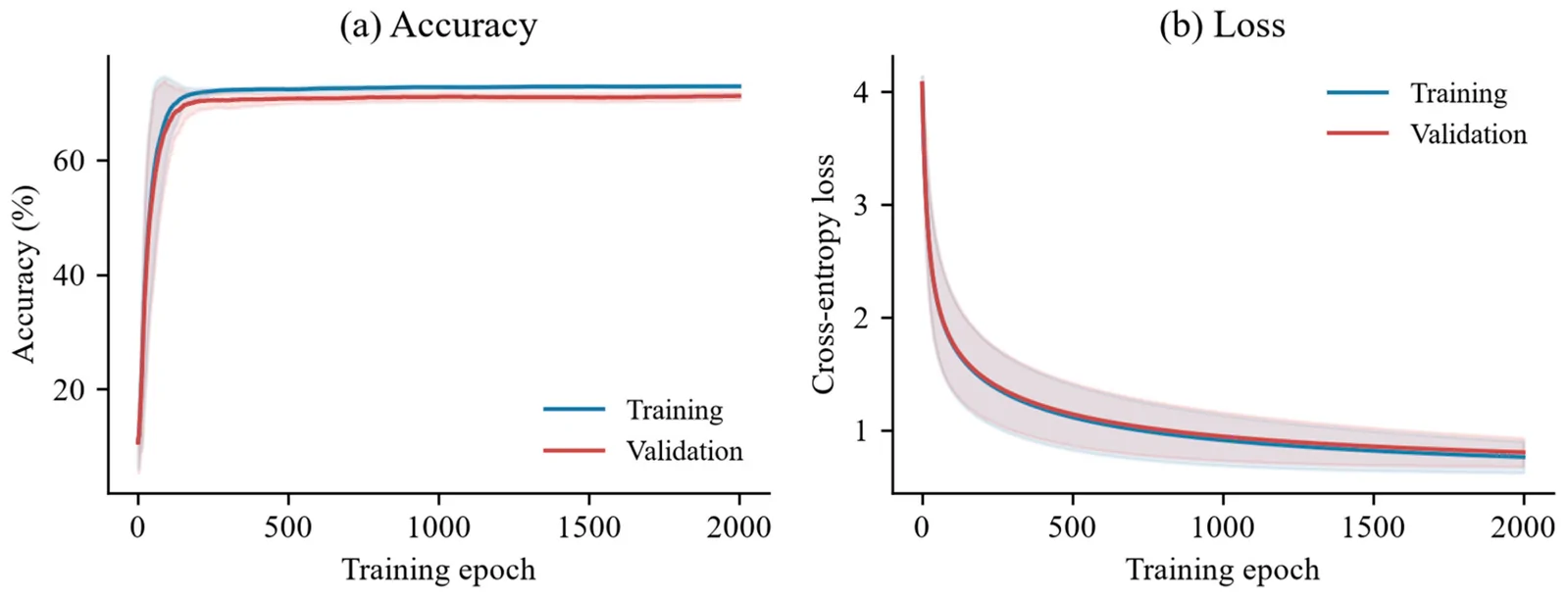
Radiation training trajectories with amplitude/noise features and a distance-scaled RBF width. Lines show means and shaded bands show ±1 sample standard deviation over seeds 2026–2028: (**a**) accuracy and (**b**) cross-entropy loss. Test trajectories are not used during training.

**Figure 7:**
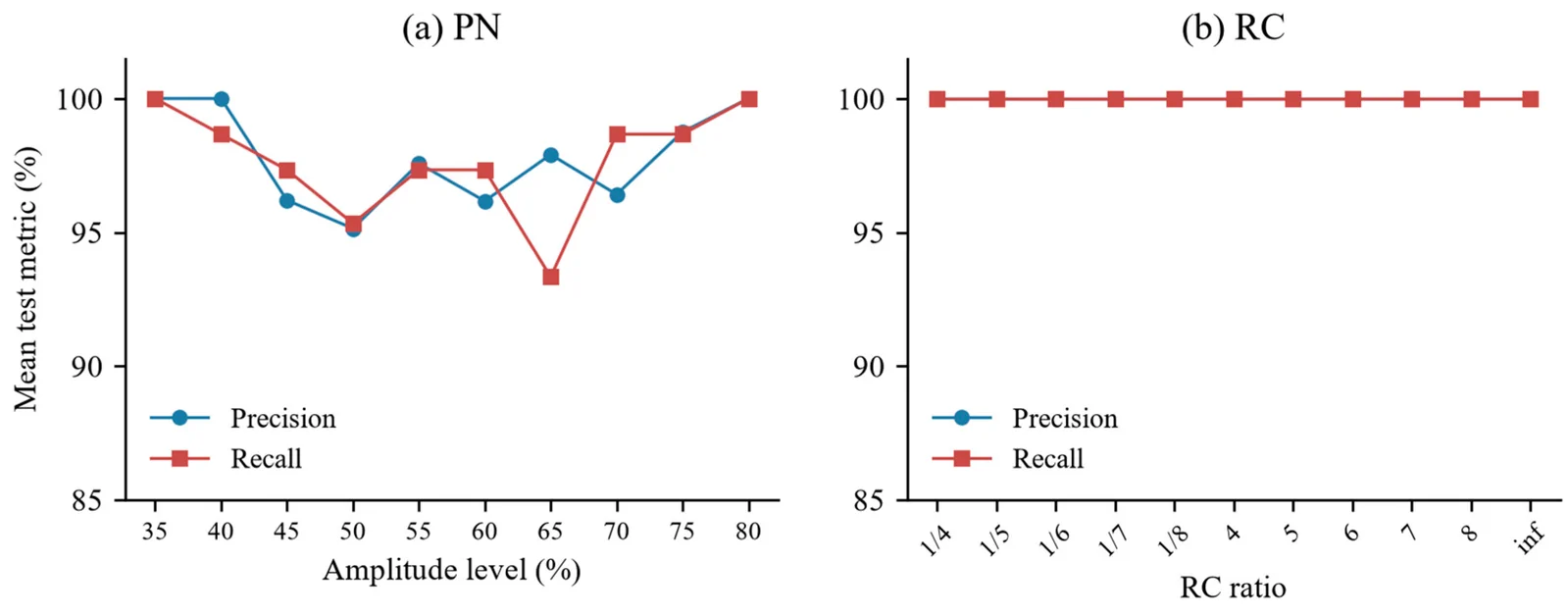
Mean classwise test precision and recall for BO + K-means RBF: (**a**) ten PN amplitude levels and (**b**) eleven RC ratios. Each mean uses ten partitions with 15 test waveforms per class per partition. The plotted quantities are precision and recall.

**Figure 8:**
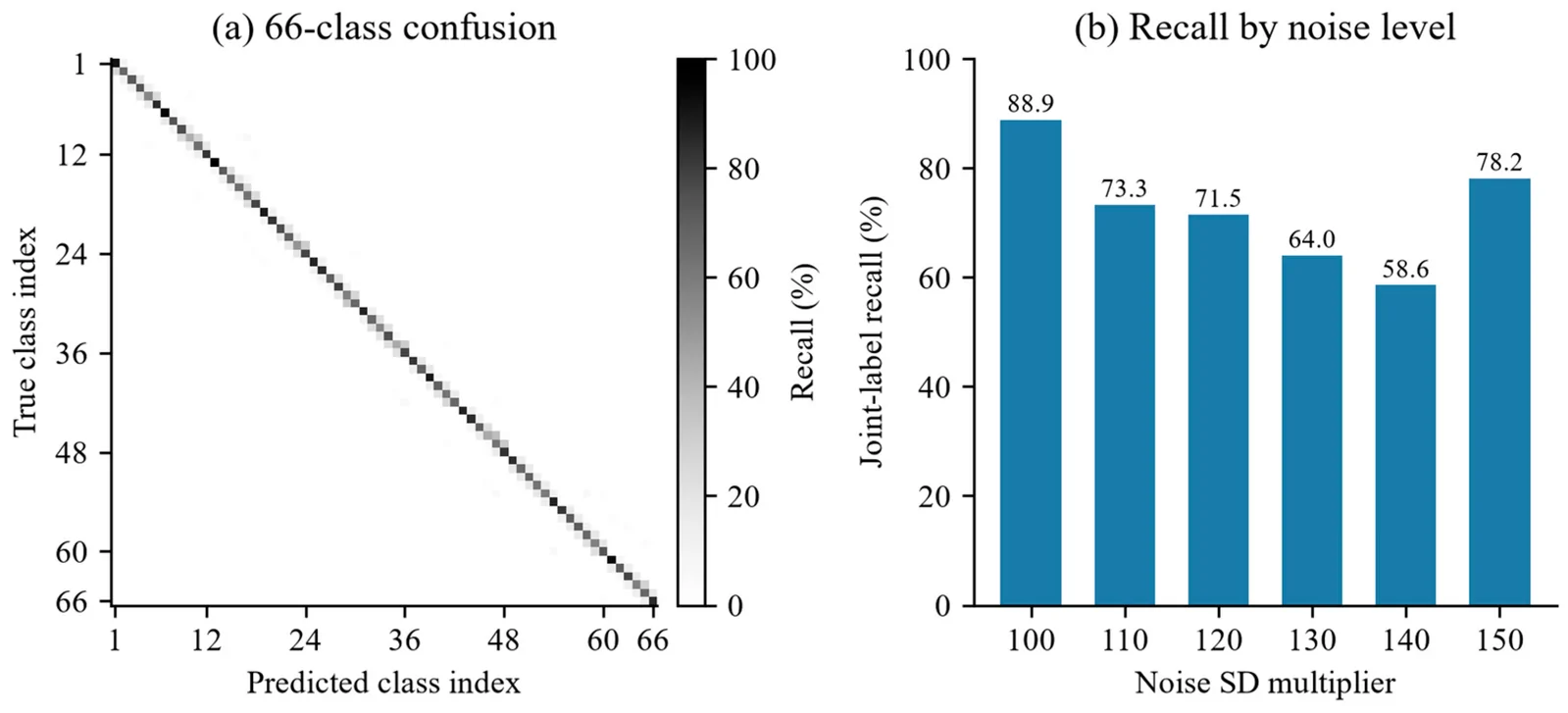
Revised radiation test results aggregated across three partitions: (**a**) row-normalized 66-class confusion matrix and (**b**) recall of the complete joint label grouped by noise multiplier. Class indices follow ascending amplitude (0.35–0.85 V) and, within each amplitude, ascending noise multiplier (100–150). Each row in (**a**) contains 45 test evaluations.

**Figure 9:**
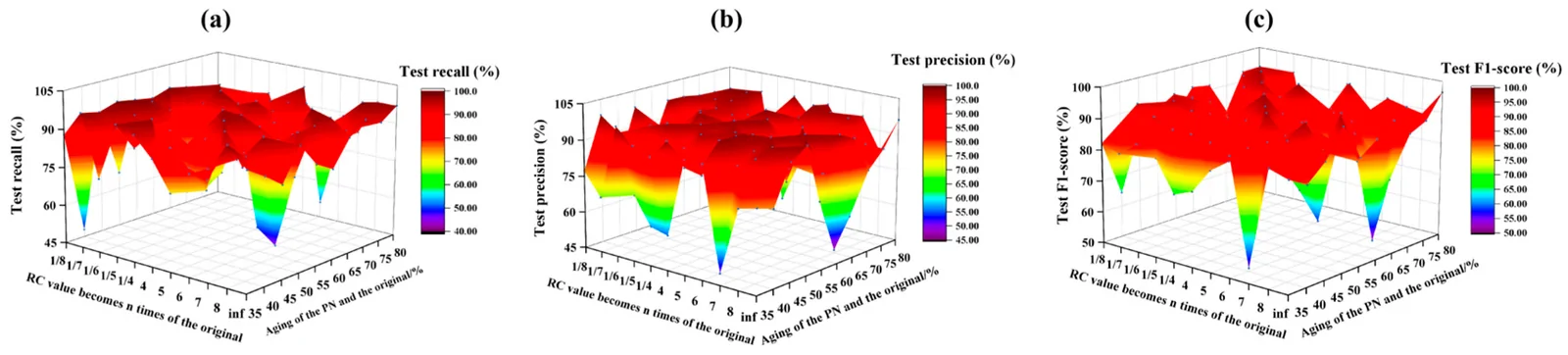
Recall, precision, and F1-score of the test set across all fault levels under the combination of PN aging and RC fault. (**a**–**c**) represent the recall, precision, and F1-score of the test set respectively.

**Figure 10:**
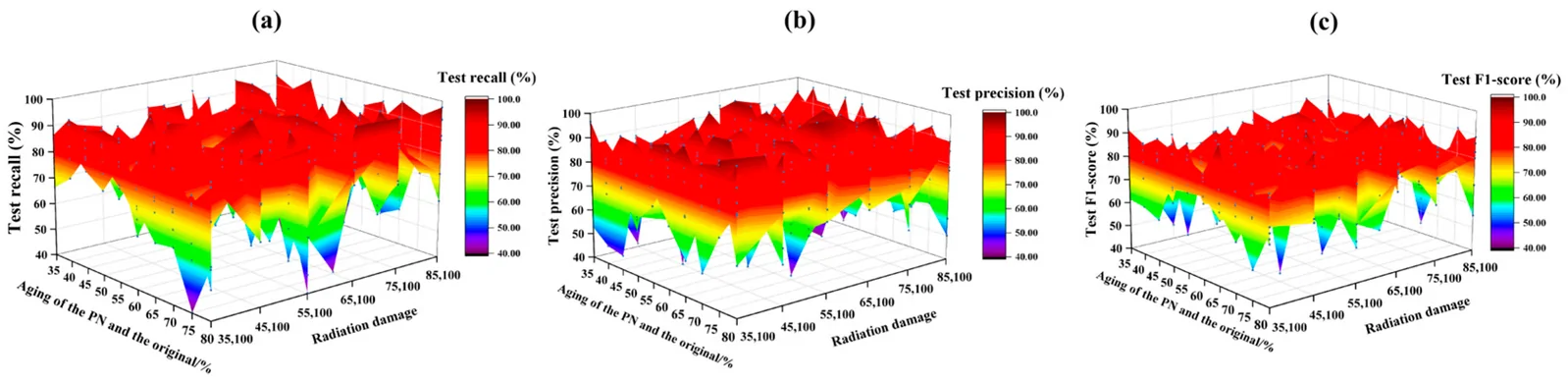
Recall, precision, and F1-score of the test set across all fault levels under the combination of PN aging and radiation damage. (**a**–**c**) represent the recall, precision, and F1-score of the test set respectively.

**Figure 11:**
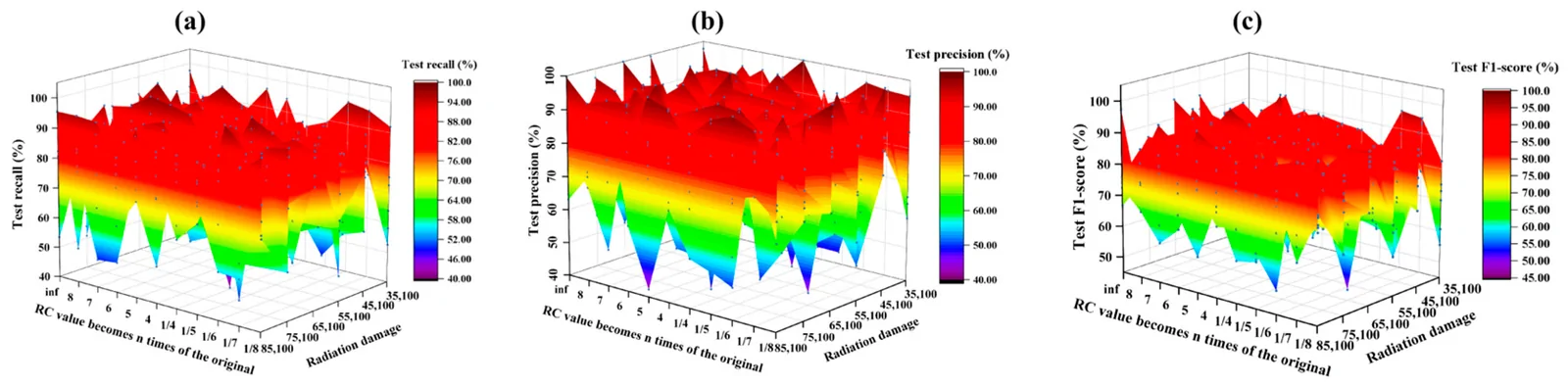
Recall, precision, and F1-score of the test set across all fault levels under the combination of RC fault and radiation damage. (**a**–**c**) represent the recall, precision, and F1-score of the test set respectively.

**Table 1:** Mean cumulative training-set explained variance (%) over ten partitions. d is the retained dimension under the 99% rule.

Task	50 PCs	100 PCs	150 PCs	200 PCs	250 PCs	d at 99%
PN	99.731	99.961	99.994	99.999	100.000	15
RC	100.000	100.000	100.000	100.000	100.000	2
Radiation	47.716	64.897	79.235	90.602	97.309	277

**Table 2:** Hyper-parameter search ranges for the evaluated RBF variants.

Parameter/Evaluation	Minimum	Maximum
Hidden centers K (all BO runs)	10	100
Absolute width σ (PCA ablation)	0.1	20
Relative width α (radiation reassessment)	0.1	3

**Table 3:** Bayesian optimization procedure used for each task and partition.

BO Procedure: 30 Evaluations per Task and Partition
1. Fit preprocessing on the training partition. Define K and width ranges. Generate five initial candidate evaluations.
2. For each candidate, reset the fit seed, fit centers and output weights on training data, and compute validation misclassification rate. Restore the search random state.
3. Update the ARD Matérn 5/2 Gaussian-process surrogate. Select the next candidate using expected-improvement-plus. Continue until 30 evaluations are complete.
4. Select the candidate with the lowest observed finite validation error. Refit with the same fixed seed and retain the epoch with the lowest validation loss. Evaluate the frozen model on the designated test partition.

**Table 4:** Selected BO + K-means parameters by split seed. PN and RC use PCA; radiation uses the revised amplitude/noise representation. Widths are in the corresponding scaled feature space.

Seed	PN: K	PN: σ	RC: K	RC: σ	Radiation: K/α/σ
2026	92	0.6917	88	0.2223	64/0.4452/0.6628
2027	100	0.6809	26	0.2334	94/0.5591/0.8337
2028	100	0.8196	92	0.4473	26/0.6594/1.0085
2029	90	0.6856	91	0.7994	Not evaluated
2030	100	0.7195	51	0.1152	Not evaluated
2031	100	0.5894	71	0.2608	Not evaluated
2032	100	0.6779	83	0.8146	Not evaluated
2033	100	0.7725	88	0.1815	Not evaluated
2034	100	0.6952	47	0.1076	Not evaluated
2035	84	0.7774	84	0.7774	Not evaluated

**Table 5:** Task-specific test results for BO + K-means RBF, reported as mean ± sample standard deviation. P and R denote precision and recall.

Task/Features	Runs	Accuracy (%)	Macro-P (%)	Macro-R (%)	Macro-F1 (%)
PN/PCA	10	97.67 ± 1.97	97.82 ± 1.84	97.67 ± 1.97	97.68 ± 1.96
RC/PCA	10	100.00 ± 0.00	100.00 ± 0.00	100.00 ± 0.00	100.00 ± 0.00
Radiation/amplitude noise	3	72.42 ± 1.57	73.05 ± 1.80	72.42 ± 1.57	72.26 ± 1.63

**Table 6:** Task test results for BO + K-means RBF, reported as mean ± sample standard deviation. P and R denote precision and recall.

Task	Runs	Accuracy (%)	Macro-P (%)	Macro-R (%)	Macro-F1 (%)
PN aging–RC	3	87.09 ± 1.52	86.40 ± 2.02	87.09 ± 1.52	85.43 ± 1.72
PN aging–radiation damage	3	77.40 ± 1.38	78.33 ± 1.45	77.40 ± 1.38	76.31 ± 1.65
RC–radiation damage	3	78.45 ± 1.28	79.25 ± 1.56	78.45 ± 1.28	77.67 ± 1.94

**Table 7:** Completed PN and RC comparisons over three matched partitions. Accuracy and macro-F1 are mean ± sample standard deviation; search and final-fitting times are mean seconds.

Task/Model	Accuracy (%)	Macro-F1 (%)	Search (s)	Final Fit (s)
PN/RBF	96.67 ± 1.76	96.68 ± 1.75	12.3	0.4
PN/Multi-SVM	100.00 ± 0.00	100.00 ± 0.00	23.8	0.5
PN/BPNN	100.00 ± 0.00	100.00 ± 0.00	61.3	3.1
PN/DRSN	100.00 ± 0.00	100.00 ± 0.00	295.1	169.9
RC/RBF	100.00 ± 0.00	100.00 ± 0.00	12.7	0.4
RC/Multi-SVM	100.00 ± 0.00	100.00 ± 0.00	33.5	0.5
RC/BPNN	100.00 ± 0.00	100.00 ± 0.00	188.1	6.5
RC/DRSN	100.00 ± 0.00	100.00 ± 0.00	205.0	201.0

**Table 8:** Mean accuracy of three frozen radiation models under specified simulated disturbances. Each condition contains 66,000 waveforms. Laplace and Student t noise preserve theoretical marginal variance; AR(1) noise preserves marginal Gaussian variance.

Simulated Condition	Mean Accuracy (%)
Gaussian control A	71.64
Control A − 20 mV baseline	20.07
Control A + 20 mV baseline	19.03
Independent Laplace noise	55.22
Independent Student t noise (df 5)	53.58
Gaussian control B	71.82
Control B signal gain 0.9	6.83
Control B signal gain 1.1	7.72
Gaussian AR(1) noise, ρ = 0.7	42.29
Second pulse, delay 50 ns, amplitude 0.2 A	1.56

**Table 9:** Computational timings for the ten-run PCA ablation (mean ± sample standard deviation). Prediction batches contain 150, 165 and 990 test waveforms for PN, RC and radiation. Feature preparation and acquisition are excluded.

Task (PCA)	Variant	Search (s)	Final Fit (s)	Test Prediction (ms)
PN	RBF	Not used	0.36 ± 0.02	0.349 ± 0.650
PN	+KM	Not used	0.38 ± 0.04	0.156 ± 0.101
PN	+BO	11.63 ± 0.52	0.38 ± 0.01	0.161 ± 0.072
PN	+BO + KM	11.98 ± 0.25	0.39 ± 0.01	0.137 ± 0.040
RC	RBF	Not used	0.38 ± 0.00	0.083 ± 0.005
RC	+KM	Not used	0.40 ± 0.01	0.085 ± 0.005
RC	+BO	12.42 ± 0.13	0.40 ± 0.01	0.101 ± 0.027
RC	+BO + KM	12.76 ± 0.12	0.41 ± 0.02	0.096 ± 0.012
Radiation	RBF	Not used	18.57 ± 6.72	2.026 ± 0.719
Radiation	+KM	Not used	18.72 ± 6.26	1.843 ± 0.476
Radiation	+BO	454.89 ± 129.82	15.70 ± 4.95	2.088 ± 0.460
Radiation	+BO + KM	516.57 ± 132.43	20.28 ± 7.08	2.299 ± 0.898

## Data Availability

Data will be made available on request.

## Abbreviations

The following abbreviations are used in this manuscript:

RBFNN	Radial basis function neural network
PCA	Principal component analysis
BO	Bayesian optimization
KM	K-means
ELM	Extreme learning machine
SVM	Support vector machine
BPNN	Back-propagation neural network
DRSN	Deep residual shrinkage network
CZT	Cadmium zinc telluride
RC	Resistance–capacitance
RMS	Root mean square
SD	Standard deviation
GP	Gaussian process
